# Silver Nanoparticles as Carriers of Anticancer Drugs for Efficient Target Treatment of Cancer Cells

**DOI:** 10.3390/nano11040964

**Published:** 2021-04-09

**Authors:** Helena I. O. Gomes, Catarina S. M. Martins, João A. V. Prior

**Affiliations:** LAQV, REQUIMTE, Laboratory of Applied Chemistry, Department of Chemical Sciences, Faculty of Pharmacy of the University of Porto, Rua de Jorge Viterbo Ferreira, n°. 228, 4050-313 Porto, Portugal; up201606016@ff.up.pt (H.I.O.G.); catsofiamartins@gmail.com (C.S.M.M.)

**Keywords:** silver nanoparticles, anticancer drugs, nanocarriers, antitumor effects

## Abstract

Since the last decade, nanotechnology has evolved rapidly and has been applied in several areas, such as medicine, pharmaceutical, microelectronics, aerospace, food industries, among others. The use of nanoparticles as drug carriers has been explored and presents several advantages, such as controlled and targeted release of loaded or coupled drugs, and the improvement of the drug’s bioavailability, in addition to others. However, they also have some limitations, related to their in vivo toxicity, which affects all organs including the healthy ones, and overall improvement in the disease treatment, which can be unnoticeable or minimal. Silver nanoparticles have been increasingly investigated due to their peculiar physical, chemical, and optical properties, which allows them to cover several applications, namely in the transport of drugs to a specific target in the body. Given the limitations of conventional cancer chemotherapy, which include low bioavailability and the consequent use of high doses that cause adverse effects, strategies that overcome these difficulties are extremely important. This review embraces an overview and presentation about silver nanoparticles used as anticancer drug carrier systems and focuses a discussion on the state of the art of silver nanoparticles exploited for transport of anticancer drugs and their influence on antitumor effects.

## 1. Introduction

Cancer is one of the most challenging diseases, characterized by the development of mutated cells that divide uncontrollably. Its pathogenesis may result from genetic deregulation or mutations that result from acute or chronic exposure to xenobiotics or environmental pollutants. Cancer cells can spread to different organs in a process called metastasis [1,2,3]. The conventional therapeutic strategies used in the treatment of cancer are chemotherapy, surgery, and radiation therapy. These therapies are used routinely based on the pathological stages and clinical signs of the disease. Despite the advances in treatment protocols, patients’ long-term survival is low and there is a high incidence of adverse effects of chemotherapy. Often, drugs used in chemotherapy have poor water solubility. Hydrophobic drugs have reduced biocompatibility and therefore need to be administered in higher dosages to achieve therapeutic concentrations. Also, low hydro solubility translates into reduced drug bioavailability and high systemic toxicity. Besides, chemotherapy drugs generally have little specificity and cause significant damage to healthy tissues and, consequently, cause adverse reactions. In addition to the problems related to the anticancer drugs addressed, early detection of cancer is also a challenge. In this sense, the combination of target-oriented drug delivery systems and controlled release can be the alternatives to overcome some limitations of conventional chemotherapy, and nanotechnology might be the solution.

Nanotechnology includes a broad multidisciplinary field that has evolved worldwide very fast over the past decade. The word “nanoparticle” comes from the Greek word “nanos”, which means “dwarf” particles. The prefix “nano” means “one billionth”. According to the American Society for Testing and Materials (ASTM) E2456-06 standard, a nanoparticle is a particle whose size is between 1 and 100 nm. However, this definition has not been strictly followed by all authors. Thus, some publications report that particles larger than 100 nm are nanoparticles (NPs) [4,5].

Recently, nanotechnology has evolved rapidly and has been applied in the most diverse domains, including in the medical field. Nanoparticles can be used in several areas, such as in tissue engineering, in the pharmaceutical, aerospace, and microelectronics industries, in production, processing, protection, and packaging of food, among others. For example, silver nanoparticles have been increasingly explored in this area due to their peculiar physical, chemical, and optical properties [6]. Silver nanoparticles (AgNPs) have intrinsic antibacterial and anticancer properties, through several mechanisms, like for example, due to silver ions or formation of radical species, released and formed respectively after the AgNPs are up took by the living cells or bacteria, which originates deregulation of important cellular mechanisms, leading to aggravated cellular damage and death. Thus, nowadays, AgNPs are already used often impregnated in antiseptic medical bandages [7], or food containers [8]. The possibility of conjugate the anticancer intrinsic property of AgNPs with the pharmacological effect of anticancer drugs can be the answer for the treatment of tumors that after a time stop responding to chemotherapy or radiotherapy. Considering the possibility of being able to use the AgNPs to carry anticancer drugs to a tumor site, and the AgNPs release the drug “in-situ” and start actuating against the cancer cells after being uptake by the cells, constitutes a promising treatment approach for cancer. Thus, the exploitation of the AgNPs as simultaneously target-directed drug delivery systems, namely of anticancer drugs, will be analyzed in this review.

The present work discusses some pedagogic concepts related to the use of general nanoparticles as drug delivery systems. Next, a scientific review is performed of the state of the art of silver nanoparticles coupled with anticancer drugs, analyzing the most relevant findings of the reviewed papers, including a detailed discussion for each drug nanoconjugate, and the chemical characteristics of the surface in the nanomaterials that enable the coupling of the anticancer drugs. Some important data of the works are compiled in a table, namely the selected drugs used for coupling with AgNPs, synthesis method and parameters, functionalization of the nanoparticles, characterization methods for monitoring of the assembly, and the results obtained in in vitro anticancer assays.

## 2. Nanocarriers for Drug Delivery

Oral and injectable are the most used routes in the administration of drugs, through conventional preparations, such as solutions, emulsions, suspensions, and solid pharmaceutical forms (tablets, capsules, etc.). However, these preparations can have in some cases limitations, namely, reduced efficacy due to the difficulty of the drug reaching exclusively its specific site of action, by circulating throughout the organism and affecting both unhealthy and healthy cells, possibly causing serious adverse effects.

Currently, nanotechnology has allowed extraordinary progress in the transport and release of drugs in specific target locations in living organisms, as well as, in a specific intended time (drug release time control) [9]. The development of nanosystems tuned for the release of a drug at a specific target location, often allows to reduce some of the adverse effects and toxicity of the carried drug. Several nanocarriers are currently being investigated [10], including organic (liposomes, dendrimers, micelles, among others) and inorganic nanoparticles (NPs), such as magnetic, silver NPs, gold NPs, quantum dots, etc. In addition to organic and inorganic NPs, there are also nanoparticles of a hybrid nature, such as NPs with an inorganic core surrounded by an organic material [11,12,13].

A targeted drug delivery system must allow to control the fate of the drug in the body, protecting the cells and tissues that are not the target of the therapy. These drugs’ nanocarriers are endowed with optimized and well-defined physical, chemical, and biological properties to efficiently improve their cellular uptake, or of the drug in relation to larger molecular structures [14]. Additionally, the possibility of controlling the size, surface charge, and surface chemistry of the nanoparticles acting as carriers, as well as the release of pre-loaded drugs at a specific site, allows to overcome other limitations of conventional therapies, namely, the need for higher dosages, low bioavailability, and chemical instability of the administered drug [15]. If the nanocarriers are designed and produced to successfully accumulate on the target, there will be a lower incidence of systemic adverse effects and a better therapeutic efficacy. Nowadays, the production of nanoparticles tailored with pre-defined physical-chemical properties allowed to adapt the drug-delivery nanoparticle to a specific cancer and to different types of anticancer drugs, since each type of cancer has unique biological expressions [16,17].

Each nanocarrier transports more than one molecule of a drug, thus allowing to increase the concentration of the drug successfully delivered to the target tumor, while at the same time without bringing consequences for healthy tissues. It is also possible to transport different anticancer drugs per NP, leading to a synergistic anticancer effect. This allows to reduce the concentration of each drug, avoiding toxicity and the development of resistance of the tumor to the chemotherapy. Yet, the NPs have limited drug loading capacity [13,18].

The nanoparticles drug delivering systems can be administered in several ways, including oral, nasal, parenteral, intraocular, among others, but systemic administration is the typical method used. It is possible to choose one route over another, to optimize patient compliance or reduce manufacturing costs [13,19].

Despite the several advantages mentioned, the NPs have some limitations that must be overcome before being used routinely in the clinic or being commercialized, especially for targeted delivery of cancer therapeutics [20]. For example, considering nanoparticles tailored for passive nanodelivery (through the Enhanced Permeability and Retention effect), only less than 1% effectively reaches the target. Losses are attributed to random distribution through the tumor and adjacent tissues, defensive actions of the mononuclear phagocytic system, or difficulties breaking through physician biological barriers. To overcome some of these difficulties, active cellular nanodelivery strategies allow higher affinity to specific ligands on target, and hence, increased probability for cellular uptake. But even active targeting strongly depends on passive diffusion throughout the organism until the target tumor tissues. In addition, the passage of nanosystems carrying drugs through the blood-brain barrier still constitutes a generalized challenge [21]. Also, generally the nanotherapeutic agents still have many limitations, including, among others, low bioavailability and the consequent use of high doses to compensate the low amount of nanocarriers reaching successfully the target, which consequently causes serious adverse effects. More information about this topic, including a review of past clinical trials with the nanocarriers for cancer therapeutics can be encountered in the work of Rosenblum et al. [20]. For these reasons, the development of strategies that minimize the described limitations becomes relevant, and hence, more studies in this field are imperative.

## 3. Silver Nanoparticles

Metal nanoparticles, such as silver, gold, and platinum NPs, are generally small, about 50 nm and have a high surface area. They have facility to cross capillaries in tissues and cells due to their reduced size. The high surface area they provide together with the possibility to chemically modify their surfaces (tunable surface chemistry), allow them to carry a reasonable number of drugs.

In this sense, metal NPs have been exploited for the controlled release of drugs in the treatment of cancer [22]. Despite it being desired that these metals are inert, there may be bioaccumulation and toxicity. On the other hand, one major advantage of metal NPs is their ability to efficiently absorb energy in the form of light and convert it to heat. Therefore, some can be used in hyperthermic tumor therapy, in which photo stimulation provides thermal energy, to make this therapy highly specific [3,5].

Among several metal NPs, silver nanoparticles (AgNPs) have been increasingly used in the pharmaceutical, aerospace, microelectronics, food industries, and in medicine due to their unique chemical, physical, and biological intrinsic properties. Their optical, biological characteristics, and high electrical conductivity can be highlighted [2]. The AgNPs proved to be useful in industrial and health products, such as coating of medical devices [23], biosensors [24], in bio-imaging [25], as antibacterial [8], antifungal [26], antiparasitic [27], antiviral [28], anticancer agents [29], and as drug carriers [30], among other applications. Silver nanoparticles have been extensively studied and recognized as useful tools in therapeutics [22], since they have a high surface/volume ratio, ease of synthesis, tunable surface chemistry and surface functionalization, and good penetration and traceability in the organism. Despite the fact that AgNPs are extensively applied in in vitro studies using different cancer cell models (due to their intrinsic anticancer effect), the exploitation of AgNPs coupled with anticancer pharmaceutical drugs has been the objective of some recent scientific studies, searching for increased antineoplastic efficiency, particularly when also used synergistically with natural anticancer products used in their synthesis (Green-Chemistry approaches) [31].

The mechanisms by which AgNPs have intrinsic anticancer activity are well addressed in several works in the scientific literature (for example, in the reviews of Morais et al. or Chugh et al. [31,32], respectively), and thus, will not be discussed in this review.

### 3.1. Synthesis Methods

In general, silver nanoparticles can be synthesized by chemical, physical, or biological methods [33]. Some of these approaches are considered to follow most of the principles of Green Chemistry for nanoparticle synthesis [34,35].

The chemical methods are based on the chemical reduction of Ag^+^ ions [36] by organic or inorganic agents, like sodium borohydride, sodium citrate, sodium ascorbate, N,N-dimethylformamide, polymers, among others. The reducing agent leads to the formation of metallic silver (Ag^0^), which agglomerates forming oligomeric aggregates. These give rise to colloidal metal silver particles. Capping agents and surfactants as stabilizers can be used, as chitosan, cellulose, and diverse polymers (polyethylene glycol (PEG), polyvinylpyrrolidone (PVP), polymethacrylic acid (PMAA)), to avoid excessive agglomeration and assisting in controlling the size of the AgNPs. The AgNPs stabilization in suspensions can be achieved through electrostatic or steric repulsion between NPs. Electrostatic stabilization is usually achieved with anionic species, such as citrate, halides, carboxylates, or polyoxoanions, which coat the AgNPs and give a negative charge to their surface. It is also possible to give a positive charge to its surface using polyethyleneimine (PEI), for example. These charging-coatings can be monitored in the AgNPs through the measurement of the zeta potential. The steric stabilization can be achieved through the interaction of AgNPs with bulky molecular groups, such as organic polymers and alkylammonium cations [37,38].

The most relevant physical methods are based on the evaporation-condensation and laser ablation techniques. Both methods do not use chemical reagents that can be dangerous for the environment and for the human organism. However, they require specialized and very expensive equipment [37].

Biological methods [39] do not use chemical reagents that can be to some extent toxic. They are based on the use of bacteria, fungi, algae, and plants that contain reducing agents that reduce Ag^+^ ions. The synthesis of AgNPs through plants is gaining popularity, since it is ecological, economical, accessible, and simple to perform. As one can imagine, thousands of different natural sources can be used, and as examples, some extracts used are derived from plants such as *Azadirachta indica*, *Eucalyptus procera*, *Calliandra haematocephala*, *Madhuca longifolia*, among others. The rich composition of plant extracts in chemical compounds has a complex activity, since it can contain, in addition to reducing and stabilizing agents, substances that coat the AgNPs (capping agents), inadvertently or intentionally. Most of the plant extracts used to synthetize AgNPs possess some sort of therapeutic activity (anti-inflammatory, anticancer, antioxidant, etc.), which can be exploited together with the intrinsic biological action of AgNPs, related with, for example, the disintegration of AgNPs in silver ions that interfere with diverse cellular processes. Thus, when using biological natural products as raw materials for AgNPs synthesis, the use of additional stabilizing or capping agents is often not necessary [33,37,40,41].

### 3.2. Characterization Methods

There are several properties and features of the nanoparticles and nanocarriers often monitored for characterization purposes, namely size, shape, morphology, particle size distribution, ultraviolet-visible spectrum (UV-Vis), zeta potential, infrared spectrum, surface area, entrapment efficiency, and drug loading capacity [33].

One of the most important characterizations of the AgNPs is the particles’ distribution size. It influences the stability of the AgNPs directly related with their physical-chemical properties, and can define the loading and release capacities of drugs. The AgNPs’ sizes determine their biological fate in vivo, distribution, toxicity, and targeting ability of the nanosystems [42]. The size of NPs is one of the factors used to predict the circulation time inside living organisms. In healthy tissues, nanoparticles are removed from the circulation by the glomerular filtration system (NPs below 20 nm in diameter are rapidly cleared by renal excretion) and NPs larger than 200 nm in diameter are easily cleared from the reticuloendothelial system (RES). After a systemic administration, NPs can accumulate in the spleen owing to mechanical filtration and then are removed by the RES. The primary mechanism of removal by the RES is through endocytosis. The interaction of macrophages with targets, like AgNPs, is dependent on their size, as demonstrated by the work of Doshi et al. [43]. Approximately, 100 to 200 nm is considered the ideal size for nanocarriers, but research has also indicated ~50 nm nanoparticle diameters to prolong systemic circulation and prevent unintended elimination, since they are large enough to prevent filtration in the spleen and uptake in the liver. Particles with a diameter of less than 5 nm are quickly removed from the circulation by renal clearance, for example. However, particles with a diameter greater than 15 nm accumulate in several organs, namely in the liver, spleen, and bone marrow. Additionally, a smaller nanoparticle size promotes an increase in the surface area, solubility, dispersion, dissolution rate, and the oral bioavailability of the drug. Regarding the latter advantage, Biswas et al. [44] synthesized valsartan loaded mesoporous silica nanoparticles which showed to be capable of improving the oral bioavailability of the drug. Furthermore, a negative surface charge is associated with a rapid removal of NPs from the blood compared to the existence of a neutral charge [45].

Usually, it is measured using scanning electron microscopy (SEM) or transmission electron microscopy (TEM). The hydrodynamic diameter measurement can be performed by the dynamic light scattering (DLS) technique [33]. The nanoparticle’s shape can be spherical, cylindrical, flat, conical, tubular, or irregular, and can also be determined using electron-based microscopy techniques.

But UV-Vis spectrophotometry is a technique that, in some cases, give valuable information about the approximate size and shape of the NPs [2,13], and it is by far the most worldwide available spectroscopy method in any research laboratory. Silver nanoparticles have unique optical properties, absorbing and scattering light efficiently, that cause them to interact with specific wavelengths of light. In AgNPs, the conduction band and the valence band are very close, with the electrons moving freely. These free electrons originate a surface plasmon resonance (SPR) band, due to the collective oscillation of the AgNPs electrons in resonance when they are excited by light at specific wavelengths. The wavelength at maximum peak absorbance (λ_max_) corresponds to the surface plasmon resonance (SPR) band. This value reflects the average size of the AgNPs’ size distribution, and usually for AgNPs is in the range 400–450 nm [33]. The AgNPs absorption properties depend on the particle size, shape, and the local refractive index near the particle surface. Thus, UV-Vis spectrophotometry is a very useful technique for the characterization of NPs and for monitoring their synthesis [2]. Normally, scientific works involving the synthesis of AgNPs, contain a thorough characterization of the nanomaterials by these techniques, TEM, DLS, and UV-Vis spectrophotometry. For example, He et al. [46] synthesized AgNPs through an eco-friendly approach, using the fruit aqueous extract of Chinese herbal *Cornus officinalis.* The as-synthesized AgNPs showed a λ_max_ of 411 nm, the DLS spectrum indicated a narrow distribution of the nanoparticles with a diameter of 12.8 ± 3.5 nm, while the TEM analysis revealed that the nanoparticles presented a quasi-spherical shape, and the particle size was 11.7 nm, close to the obtained DLS value.

In addition to assessing the size and shape of NPs, the measurement of the zeta potential is relevant, it gives information about the stability of the particles and the electrostatic repulsion between them. If the zeta potential is superior to (+) 25 mV or less than (−) 25 mV, the NPs are considered stable in suspension and are unlikely to aggregate [33,47]. When Elamawi et al. [48] synthesized AgNPs using *Trichoderma longibrachiatum,* a negative zeta potential value was achieved, indicating the repulsion among the as-synthesized AgNPs and the increase of the stability of the formulation.

The Fourier-transform infrared spectroscopy (FTIR) technique can also be performed to ascertain the functional chemical groups associated with the AgNPs, derived mainly from their capping or stabilizer agent [47]. Huq [49] synthesized AgNPs by a green chemistry approach, using *Pseudoduganella eburnea* MAHUQ-39. The functional groups at the surface of the AgNPs were identified with a FTIR analysis.

As for the characterization of the nanocarriers, the entrapment efficiency is a very important physicochemical attribute since it reflects the percentage of the drug that is successfully entrapped or adsorbed in the NP [50]. It is determined by the difference between the total amount of drug used to prepare the nanoparticles and the amount of free drug present in the aqueous medium. This value is divided by the total amount of drug used and multiplied by 100 to obtain the percentage. In addition to this attribute, the loading capacity, which corresponds to the amount of drug-loaded per unit weight of the nanoparticle, can also be evaluated [51].

### 3.3. Biotoxicity

Although nanotechnology has become increasingly important today, there is still reduced and confirmed information about the risks of exposure of humans, animals, and environment to NPs, particularly those of silver, in terms of toxicity at short and long term [37,52]. The synthesis of NPs may presuppose the use of substances that are naturally toxic to living organisms or the environment. Most studies address these effects only when they are administered by inhalation, damaging the respiratory tract. The toxic effects are mostly studied in animal models, where metal NPs have shown an increase in the production of radical species and disruption of platelet function [2,3,13], for example.

There are in vitro studies that concluded that AgNPs are toxic to several organs, such as the lungs, liver, brain, vascular system, and reproductive organs. In this scope, AgNPs would induce the expression of genes involved in cell cycle progression and apoptosis. Possible mechanisms of toxicity include inducing the formation of reactive oxygen species (ROS) and oxidative stress, thus leading to DNA damage and apoptosis [53]. De Matteis et al. [54] concluded that the toxicity in cells treated with AgNPs is mainly caused by the release of Ag^+^ ions in the cytosol, after internalization of AgNPs through endocytosis and their dissolution in acidic environment. Hence, the oxidative stress, DNA damage, and cell death verified in the presence of AgNPs are mainly due to the impairment of physiological metabolic and cell cycle mechanism by silver ions present in the cytosol. The activation of metallothioneins and the prevention of cytotoxicity using Ag^+^ chelating agents strengthen this hypothesis. On the other hand, Suarez et al. [55] focused on understanding physiological disruptions caused in hepatic cells by exposure to very low doses of silver nanoparticles mimicking chronic exposure. The authors found that there was a rapid entry of soluble silver ions into the nucleus, where it accumulates and impairs nuclear receptor activity, which is detrimental to liver metabolism.

There are also some in vivo studies focused on the cytotoxicity and genotoxicity of AgNPs. Due to their small size, they have high mobility and humans are easily exposed through the skin, inhalation, ingestion, etc. AgNPs can travel from the site of exposure to other organs easily, penetrating cells [37]. In the work conducted by Lee et al. [56], it was revealed that exposure to AgNPs modulates the expression of several genes associated with motor neuron disorders, neurodegenerative diseases, and immune function, indicating potential neurotoxicity and immunotoxicity resulting from exposure. In another work, Ahamed et al. [53] concluded that AgNPs cause reproductive failures, malformations during development, and morphological deformations in some animal models.

The genotoxicity and cytotoxicity of AgNPs are influenced by several physicochemical characteristics, such as concentration, charge, and surface functionalization, size, and shape [57]. The experimental results reported until recently are not enough to conclude with precision which are the effects and mechanisms of toxicity of AgNPs. However, toxicity is a limiting factor for its in vivo use [20,58,59].

### 3.4. Surface Modifications

The modification of the surface of nanoparticles is of great relevance, since, in addition to decreasing the toxicity of the initial stabilizing agents and AgNPs, it also prevents their aggregation and improves the ability to target specific cells, namely cancer cells [45].

Accordingly with Gali-Muhtasib et al. [5], an efficient anticancer nanocarrier must meet some requirements: (i) affinity for the anticancer drug, allowing its conjugation; (ii) exclusive release of the drug at the target site, thus presenting specificity for the tumor; (iii) the nanoparticle-anticancer drug complex must be stable in the serum; and (iv) the nanoparticles must be degraded in a way that is safe for the living organism.

**Figure 1:**
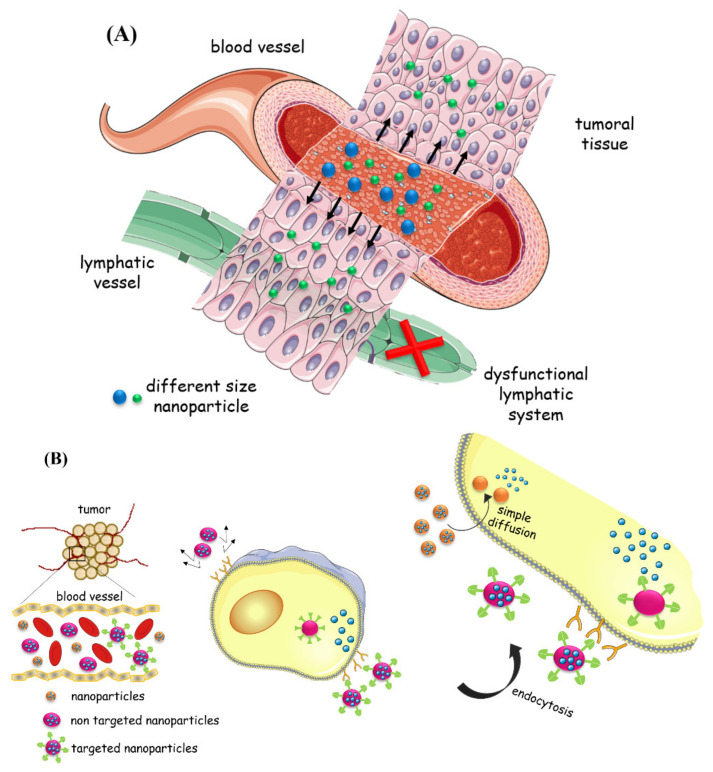
(**A**) Enhanced permeability and retention effect; (**B**) Passive (simple diffusion) and active targeting (endocytosis) of nanoparticles.

Nowadays, due to the wide range of available synthesis methods, AgNPs are relatively easy to synthesize and can be manipulated to achieve passive or active targeting [20]. They can be synthesized with specific surface ligands to promote cellular active transport, like for example polyethylene glycol (PEG)-poly lactide (PLA), chitosan, silica-based and poly(lactic-co-glycolic acid) (PLGA), or with controlled size suitable for passive transport at tumor locations. Regarding passive targeting, as tumors form a defective fenestrated vasculature, with large gaps (from 100 to 800 nm) between the endothelial cells and with inefficient lymphatic drainage near the tumor sites, nanoparticles may or may not cross these gaps in the vascular systems because it depends on their size. Smaller NPs cross these gaps and accumulate in places close to the tumor, thus reducing their exposure to normal tissues, with a consequent reduction in adverse effects. This enhanced permeability and retention effect (EPR effect, Figure 1A) results in a passive actuation of the drug to the tumor, representing another advantage over the free drug. Also, the active targeting of NPs, through the conjugation of a ligand with their surface, is more efficient. This special ligand can bind specifically to receptors or antigens that are over-expressed on the surface of cancer cells (Figure 1B). This approach improves specificity and, consequently, drug uptake and retention at the tumor site, thus reducing systemic toxicity [3,13]. Active targeting can be considered as an improvement of passive targeting, since the cellular internalization of NPs by the target cells is increased, but the tumor localization is not influenced, continuing to depend on passive diffusion.

The surface properties of NPs also influence the blood circulation time. After their administration, NPs can bind to serum proteins, called opsonins, such as immunoglobulins and complement proteins that contribute to the recognition of NPs by macrophages. In this way, opsonization is a key factor in determining whether NPs remain in the bloodstream or whether they are phagocyted by macrophages. Thus, modifying its surface is a powerful strategy to increase or decrease its circulation time in the blood [18].

There are several strategies for conjugating anticancer drugs to the AgNPs: encapsulation, entrapment, and attachment to the surface of nanoparticles [60]. For example, the incorporation of AgNPs in the structure of liposomes, improves stability, biocompatibility, and lowers toxicity [61]. In the work of Azizi et al. [62], kappa-carrageenan hydrogel beads of AgNPs were designed to allow a controlled release of silver ions.

**Figure 2:**
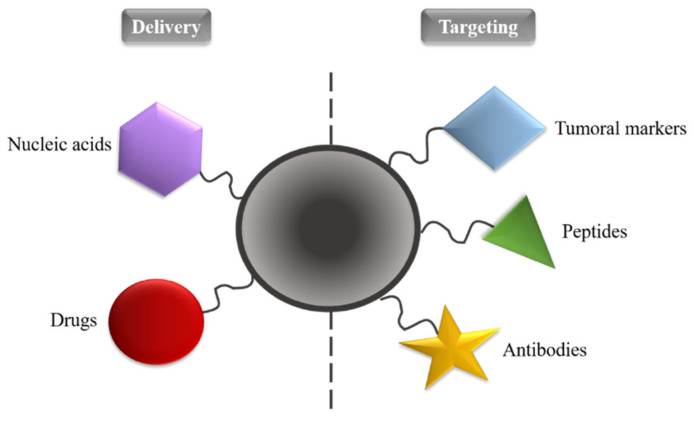
Applications of noble metal nanoparticles in cancer.

The modification of the surface of AgNPs, highlighted in this review, may be based on non-covalent or covalent interactions. Generally, the former includes electrostatic interactions and Van der Waals forces. Covalent modifications are mainly based on direct chemical bonds and linker molecules. AgNPs can be coated with peptides, like for example CSG-LL37 peptide [63], antibodies, such as immunoglobulin G [64], polymers, like polyvinyl alcohol (PVA), polyethylene glycol (PEG) and polyvinylpyrrolidone (PVP) [65], nucleic acids, such as DNA [66], and tumor markers [67] (Figure 2), thus increasing the effectiveness of target-directed drug release, and allowing its use in other applications, like in imaging techniques and target treatments with anticancer drugs, for example doxorubicin [5,68,69]. The state of the art of anticancer drugs coupled to AgNPs is discussed in following.

## 4. Anticancer Drugs Coupled to Silver Nanoparticles

With their exclusive characteristics, silver nanoparticles can be an innovative approach for cancer treatment, in two perspectives: they reveal intrinsic anticancer properties, and can be used as carriers of anticancer drugs, enabling a therapeutic of dual treatment. Regarding the latter approach, the transport systems have many advantages over free anticancer substances, which were mentioned previously. In addition to these benefits already listed, there is the fact that silver itself has anticancer activity [2,70,71,72]. For these reasons and considering that effective cancer treatment continues to be a big challenge nowadays, it is relevant to have updated knowledge about the state of the art of silver nanoparticles coupled with anticancer drugs and if it is in fact a promising approach for cancer treatment. Thus, the revision will include the obtained results with cellular assays for each case encountered in the scientific literature.

The selection of the works for the present review was based on pre-established inclusion and exclusion parameters. Only studies involving AgNPs coupled with pharmaceutical anticancer drugs were selected for this review, making hundreds of papers immediately excluded from the query in the databases. For example, studies that alluded to AgNPs combined with natural substances with associated anticancer activity were excluded, since they were not pharmaceutical drugs. Additionally, many of the works resulting from the queries were related to the intrinsic anticancer effect of AgNPs in several cell lines, which, therefore, do not suit the theme of this review by absence of the coupling with a pharmaceutical anticancer drug. Surprisingly, the thorough verification of the search results in 3 databases, retrieved a total of 11 papers, till the date of June 2020.

The parameters that were collected from each of the selected studies were related to: (i) synthesis of the nanocarriers, including synthesis method, reagents used, the ligand(s) used in the functionalization of the surface of AgNPs, temperature, time and pH; (ii) characterization of the AgNPs involving SPR band and size; (iii) the studied cancer cell lines, the cellular viability method used and the obtained results, expressed as a percentage of cell viability or as IC_50_ (concentration of the nanocarrier that causes the death of 50% of the cells, in this case). A complete compilation of the gathered data is found in the Table 1, which summarizes all the papers related to the review and facilitate the overall analysis.

**Figure 3:**
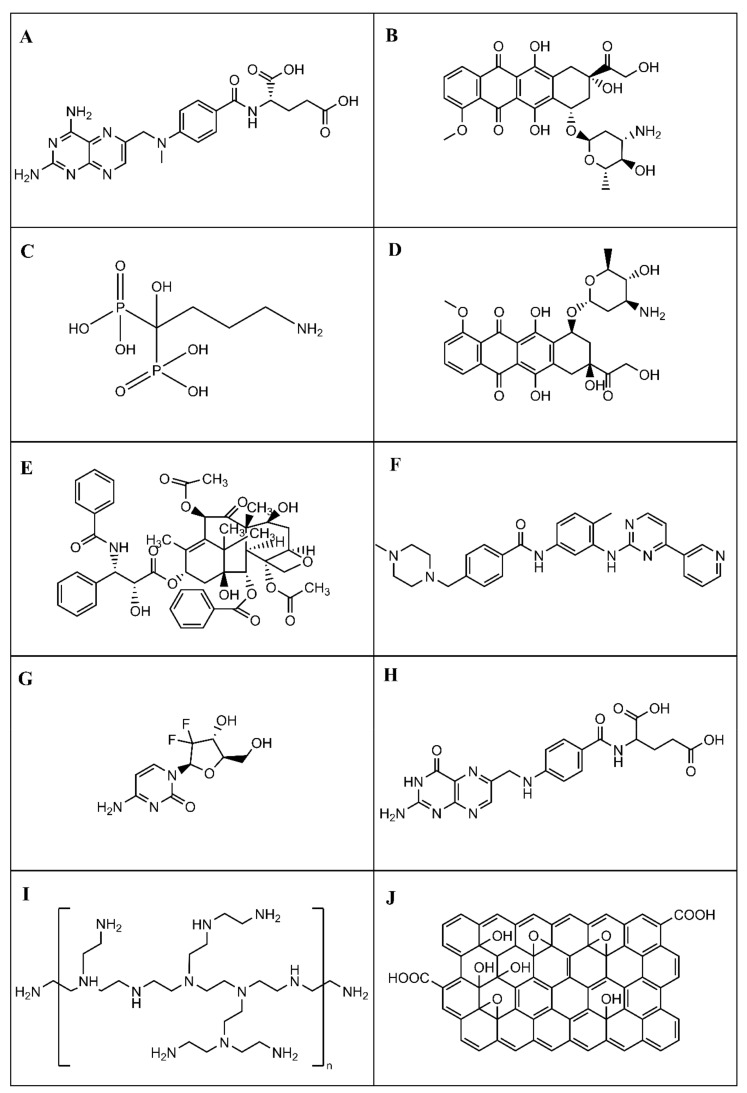
Chemical structures of anticancer drugs (**A**–**G**) and AgNPs capping agents (**H**–**J**) to mediate interaction between AgNPs and anticancer drugs. (**A**)—methotrexate; (**B**)—doxorubicin; (**C**)—alendronate; (**D**)—epirubicin; (**E**)—paclitaxel; (**F**)—imatinib; (**G**)—gemcitabine; (**H**)—folic acid; (**I**)—polyethyleneimine, branched; (**J**)—graphene oxide.

The data gathered in Table 1 were analyzed and their discussions were organized accordingly with the pharmaceutical drug coupled to the AgNPs (Figure 3). Following the literature revision, the anticancer drugs identified for coupling to silver nanoparticles were methotrexate, doxorubicin, alendronate, epirubicin, paclitaxel, imatinib, and gemcitabine. Figure 3 contains the chemical structures of those anticancer drugs. Thus, it is also presented, for each case, a detailed discussion of the chemical interactions between the AgNPs and the anticancer drugs by which the coupling is possible, enlightening the chemical interactions at the AgNPs surface. The chemical modifications used at the surface of AgNPs were also exploited in the proposed work. A special emphasis was given to the interaction nature between drug-AgNPs or drug-NPs capping. Only some of the reviewed works addressed which functional chemical groups were involved in the interaction between anticancer drugs and capping agents or AgNPs. It was found that the functional chemical groups capable of establishing interactions with the AgNPs surface were mainly the carboxyl and hydroxyl groups in their oxidized form and the amine groups. Additionally, some discussion about the synthesis method and the results obtained in the tested cell lines were also addressed.

### 4.1. Methotrexate Anticancer Drug

Methotrexate (Figure 3A) is an anionic anticancer drug that belongs to a class of medications called antimetabolites, it is an analog of folic acid and is used to treat various types of cancer, namely breast cancer and lung cancer. It has a short plasma circulation time (3–17 h) and a high rate of cell efflux, requiring high therapeutic doses (15–20 mg/m^2^, twice a week). Some side effects that this drug can cause are hair loss, decrease in the number of blood cells, liver damage, lung damage, among others [73,78,79].

**Figure 4:**
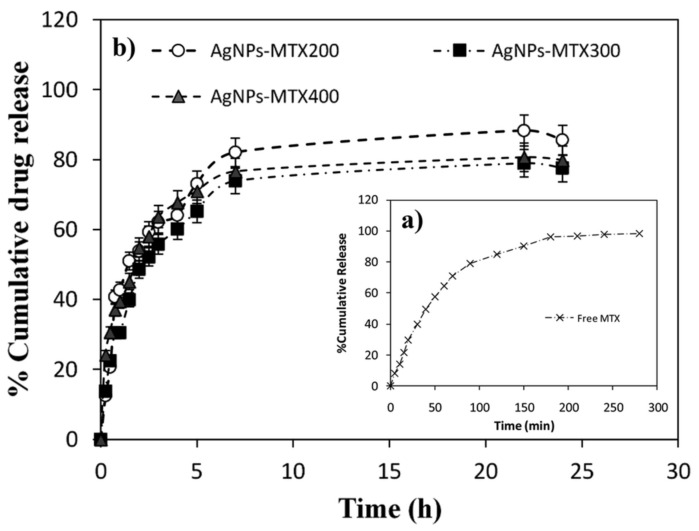
Release profiles of (**a**) free MTX, complete after 180 min; and (**b**) AgNPs-MTX 200, AgNPs-MTX 300, and AgNPs-MTX 400 (200, 300, and 400 refer to different initial MTX/Ag ratios). Reprinted from Ref. [73], with permission from The Royal Society of Chemistry, 2020.

In 2020, Rozalen et al. [73] synthesized AgNPs (mean size of 11.13 ± 2.3 nm), which were then coupled to methotrexate (AgNPs-MTX). AgNPs-MTX was obtained through chemical synthesis using sodium borohydride and citrate as reducing agents, acting citrate as also capping agent. Aqueous solutions of sodium borohydride and trisodium citrate were mixed and heated to 60 °C, in the dark and under vigorous stirring. Then, a silver nitrate solution was added dropwise. The temperature was raised to 90 °C and the pH was adjusted to 10.5, using sodium hydroxide. The solution obtained was kept under stirring for 30 min. Subsequently, the prepared AgNPs were cooled at room temperature, centrifuged at 12,000 rpm for 15 min and stored at −80 °C for 48 h for further freeze-drying. Finally, different concentrations of methotrexate solutions in potassium carbonate were added drop by drop to fresh prepared suspensions of AgNPs and the final colloids were kept under stirring for another 30 min, giving place to the formation of AgNPs-MTX 200, AgNPs-MTX 300, and AgNPs-MTX 400, according with the concentration of MTX solution. Again, the suspensions were cooled at room temperature, centrifuged at 12,000 rpm for 15 min and stored at −80 °C for 48 h for further freeze-drying. Initially, the citrate coats the nanoparticles, acting as capping, and it is then replaced by methotrexate molecules. Methotrexate also stabilizes the nanoparticles, since it confers them a negative charge as citrate does, resulting in their electrostatic repulsion. The authors confirmed by spectroscopic measurements that the MTX molecules interact with the AgNPs through covalent bonds involving the carboxylic groups of the drug, after exchange of citrate molecules at the AgNPs surface. The respective calculated drug loading capacities were 28.3, 31.48, and 40.4% *w*/*w* for AgNPs-MTX 200, AgNPs-MTX 300, and AgNPs-MTX 400. Some in vitro release studies identified differences between free MTX and MTX coupled to AgNPs, with a delay for the later in about 4 h (Figure 4). It can be observed that for free MTX, about 80% of release was achieved in 100 min, but for AgNPs-MTX, the same percentage (75–80%) was only reached upon more than 5 h.

After the assembly of AgNPs-MTX, the anticancer activity of the free drug MTX, AgNPs, and AgNPs-MTX, was evaluated against colorectal cancer cell line (HTC-116) and lung carcinoma cell line (A-549). Regarding HTC-116, the free drug MTX originated IC_50_ values at 12, 24, and 48 h of 1051, 169, and 70 µg/mL, respectively; the AgNPs originated IC_50_ values of 186, 98, and 63 µg/mL; while AgNPs-MTX 400 allowed to obtain lower values: 88, 38, and 23 µg/mL. It was evident the synergistic effect between AgNPs and methotrexate (AgNPs-MTX 400) on the HTC-116 cell line (IC_50_ = 70 µg/mL for free MTX, IC_50_ = 63 µg/mL for AgNPs and IC_50_ = 23 µg/mL for AgNPs-MTX 400, at 48 h). This means that for the same anticancer effect, a lower dose of AgNPs-MTX was required compared to free methotrexate. Additionally, the authors asserted that the anticancer effect was more pronounced in the colon cancer cell line (HTC-116) than in lung cancer cell line (A-549) since the latter does not have folate receptors. There is a greater uptake of these nanosystems by cancer cells that have folate receptors, such as the HTC-116 cellular line, than those that do not, since methotrexate mimics folic acid, thus competing for the same receptors. Several cancer cells, namely the HTC-116 cancer cell line and the breast cancer cell line MCF-7, over-express folate receptors, which have a limited distribution in normal tissues [80]. In the Table 1, for the A-549 cell line, it can be confirmed the IC 50% for AgNPs-MTX as ≈86%, ≈79%, and ≈32%, for 12, 24, and 48 h, respectively. While for the AgNPs only, the values were ≈96%, ≈85%, and ≈84%, for 12, 24, and 48 h. In, fact, the AgNPs exerted less cytotoxic effect in the A-549 cell line than in HTC-116 cell line (89% at 12 h and 59% at 48 h), and the same when using AgNPs-MTX (69% at 12 h and 36% at 48 h, for the HTC-116 cell line).

A toxicity test was carried out on zebrafish, in which the free MTX exhibited accentuated toxic effects, in accordance with increasing embryonic mortality rate (13−20%), phenotypic changes or pericardial edema. However, the conjugate AgNPs-MTX showed less toxicity: light pericardial edema, inclusively disappearing at 48 h, and lower mortality rate (less than 5%). The results of the work seem to indicate a reduction in the systemic toxicity of the nanosystem in relation to the use of free MTX. Therefore, considering the synergistic effect of AgNPs-MTX and the lower cytotoxicity caused by this nanosystem, the inclusion of AgNPs as drug carriers of methotrexate in the treatment of cancer should be further exploited, including other cancer cell lines.

**Figure 5:**
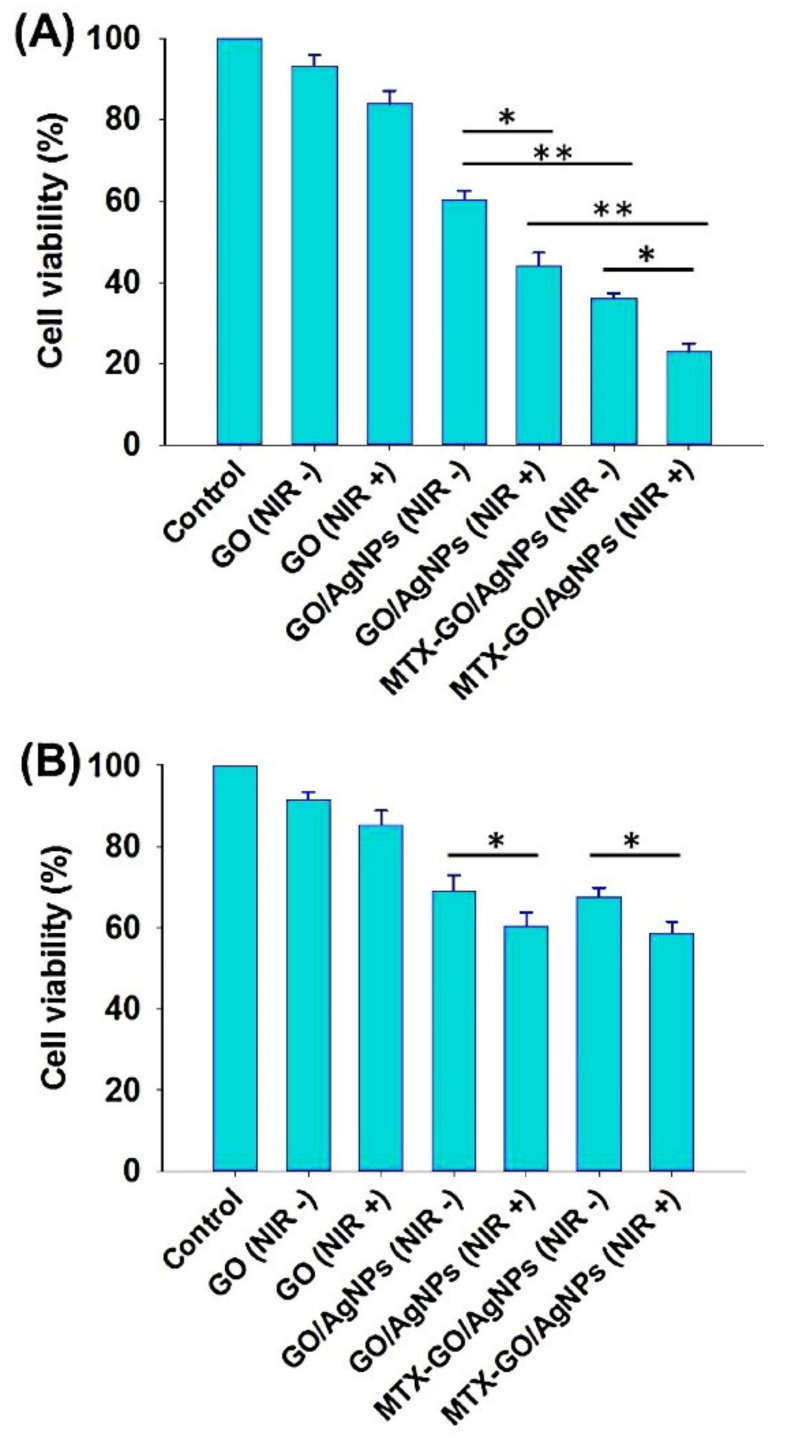
Cell viability studies of cancer cells MCF-7 (**A**) and HepG2 (**B**) upon incubation for 48 h with MTX-GO/AgNPs, and other nanocomposites, with and without NIR (808 nm NIR laser, 3.0 W/cm^2^, 5 min), through MTS assay. Statistical significance analyzed at different levels: * *p* < 0.05, ** *p* < 0.01. Reprinted from Ref. [30], with permission from Elsevier, 2021.

In 2017, a nanocomposite of AgNPs embedded in graphene oxide (GO) and coupled with methotrexate (MTX-GO/AgNPs) was produced by Thapa et al. [30] (Table 1) through chemical synthesis. GO-based materials provide the advantage of using near infrared radiation (NIR) for photothermal ablation of tumor cells. First, GO was synthesized using graphite, sulfuric acid, and potassium permanganate. Graphite was stirred with sulfuric acid for 3 h, and then a small amount of potassium permanganate was gradually added, followed by the fast addition of another amount of potassium permanganate, under a controlled temperature (<20 °C). The mixture was stirred at 35 °C for 30 min, followed by the addition of water and heating to 98 °C for more 30 min. To terminate the reaction, a distilled water:hydrogen peroxide (3:2) mixture was used. Repeated washing and centrifugation with hydrochloric acid and distilled water was performed until neutral pH. The resulting product was homogenously mixed in distilled water and probe-sonicated to obtain GO of approximately 100 nm. Subsequently, GO/AgNPs were synthesized by preparing two different dispersions of GO: (i) adding silver nitrate to a GO dispersion; and (ii) adding glucose as reducing agent, and starch, as the stabilizer to prevent agglomeration of AgNPs once formed. Both dispersions were heated to 80 °C and the latter was added to the former under vigorous stirring. The temperature of the resulting mixture was maintained at 80 °C for 4 h. The synthesized GO/AgNPs were centrifuged and repeatedly washed with deionized water. Finally, to produce MTX-GO/AgNPs, a solution of ethyl-N′-(3-dimethylaminopropyl) carbodiimide hydrochloride (EDC) was used to activate the carboxylic acid groups of graphene oxide, to react with the methotrexate amines. EDC reacts with carboxylic acid groups to originate an intermediate that can be displaced by nucleophilic attack from primary amino groups. The primary amine forms an amide bond with the original carboxyl group, and an EDC by-product is released [81]. The mixture of EDC and GO/AgNPs was stirred for 30 min at room temperature. Then, the MTX solution was added, and the resulting mixture was heated to 50 °C and stirred for 24 h, Repeated washing and centrifugation originated the MTX-GO/AgNPs formulation. Also, polyvinylpyrrolidone K30 (PVPK30) served as a coating for MTX-GO/AgNPs to avoid the aggregation of graphene oxide in physiological fluids. The FTIR analysis of MTX-GO/AgNPs suggested that the AgNPs interacted with the functional groups of GO that contain oxygen (Figure 3J), due to the reduction of the intensity of peaks at 1700 and 3400 cm^−1^ (correspondent to OH groups and stretching vibrations of C=O carboxylic moieties). Methotrexate forms a covalent bond with GO/AgNPs, through an amide bond, between a carboxylic acid of graphene oxide and an amine group of the drug. Considering other works in the literature, MTX-GO/AgNPs are especially useful in tumors that overexpress folate receptors, since methotrexate is an analog of folic acid, as previously mentioned. In the work, the authors did not test normal cell lines but conducted studies in the MCF-7 and HepG2 cancer cell lines. The breast cancer cell line MCF-7 overexpresses folate receptors, and hence, the cell viability obtained was about 22% (approximate deduction in Figure 5A), when treating MCF-7 cells with MTX-GO/AgNPs and applying a radiation with an 808 nm NIR laser (3.0 W/cm^2^) for 5 min. Without the assistance of NIR, the cell viability was roughly 38% (again, by approximate deduction in Figure 5A). In Figure 5A is evident the potentiating cytotoxic effect when adding AgNPs to GO in the anticancer treatment of MCF-7 cells, and even further when adding MTX to the previous formula.

As already mentioned in the previous work, with AgNPs conjugated to methotrexate it was possible to increase their uptake by cancer cells and decrease the systemic toxicity of this drug and overcome resistance problems associated with it. In cancer cells that do not overexpress the folate receptor, such as the HepG2 hepatocarcinoma cell line, the nanosystem showed lesser cytotoxicity, with only about 58% cell viability (approximate deduction by Figure 5B) when using NIR with MTX-GO/AgNPs. The production of reactive oxygen species in both cell lines was also evaluated. MTX-GO/AgNPs intensified the production of ROS only in the MCF-7 line compared to AgNPs, due to the greater uptake mediated by the folate receptors by these cells. When folic acid pretreatment was used, the generation of ROS decreased, which proves the fundamental role of folate receptors in cellular uptake of MTX-GO/AgNPs and consequent production of ROS. In addition, a Western blot assay of MCF-7 and HepG2 cells was performed following treatment with GO/AgNPs, MTX-GO/AgNPs, and MTX-GO/AgNPs with NIR. There was a greater expression of pro-apoptotic proteins, proteins related to cell cycle arrest and apoptotic protein c-caspase 3 in MCF-7 cells treated with MTX-GO/AgNPs than in HepG2 cells. NIR irradiation enhanced the expression of c-caspase 3 in MCF-7 cells. In conclusion, this nanocarrier combines the synergistic effect of AgNPs, which increases the production of reactive oxygen species responsible for damaging DNA, leading to apoptosis of graphene oxide, which by absorbing near infrared radiation can be used for photothermal ablation of tumors, and of methotrexate, which has anticancer pharmacological activity. Nonetheless, it would have been relevant to perform cytotoxicity tests on normal cells, as well as to compare the anticancer activity of MTX-GO/AgNPs and free methotrexate.

### 4.2. Doxorubicin Anticancer Drug

Doxorubicin [82] (Figure 3B) is an antibiotic of the anthracycline family that is widely used to treat some cancers, namely breast and ovaries cancers, multiple myeloma, among many others. However, it has serious adverse effects, such as cardiac muscle injury. Thus, given its accumulation in various organs, such as heart, liver and lungs, a nanosystem that directs the drug to the target site would be of great interest, thus reducing adverse effects [70].

Also using graphene oxide, in 2019, Palai et al. [70] synthesized AgNPs coupled with doxorubicin (NGO-AgNPs-PEG), by exploiting a biosynthesis-based approach. Firstly, the NGO was synthesized using concentrated sulfuric acid, potassium permanganate, and an aqueous solution of hydrogen peroxide. The aqueous suspension of NGO was then stirred up to 12 h, ultrasonicated for 30 min, filtered, and freeze dried. The aqueous extract of the leaves of *Azadirachta indica* was prepared from washed and dried leaves of *A. indica* and ultrapure water through distillation at 100 °C for 15 min under continuous stirring. The cooled filtered extract was centrifuged at 7000 rpm for 15 min and stored in an airtight container at 4 °C. The compounds of the extract were used as reducing and stabilizing agents, yet the authors did not include an analysis regarding the main chemical composition of the extracts. A silver nitrate solution was mixed with the colloidal dispersion of NGO, by ultrasonication for 1 h. This mixture was sonicated for another 30 min. After, the aqueous leaf extract was added drop wise under vigorous stirring at room temperature for 1 h, giving rise to silver decorated NGO nanocomposites, which were dried by lyophilization after ultra-centrifugation. The interaction between the AgNPs and NGO occurs through the non-reduced hydroxyl and carboxyl groups at NGO surface. High-resolution transmission electron microscopy revealed that the AgNPs formed at the NGO material had sizes less than 25 nm. Interestingly, AgNPs produced without NGO were shown to have sizes about 30−35 nm, indicating that NGO influences the process formation of AgNPs in the NGO matrix. However, during the reduction process of ionic silver, partially reduced graphene oxide is more prone to agglomeration, unless it is stabilized by polymers through functionalization. Thus, graphene oxide was amino-pegylated, through covalent bonds, using the EDC reagent to activate the GO carboxylic acids. As mentioned before, EDC works by activating carboxyl groups for direct reaction with primary amines through amide bond formation. Polyethylene glycol (PEG) is a biocompatible polymer capable of reducing opsonization and uptake by the reticuloendothelial system, thus increasing the circulation time of graphene-based nanocomposites in the bloodstream. So, the functionalization by this polymer decreases toxicity of nanomaterials in healthy cells. Following this reasoning, amino-PEG was mixed with NGO-AgNPs composites and the resulting mixture was ultrasonicated for 1 h. Then, EDC and N-hydroxy succinimide were added to the mixture under vigorous stirring for 12 h. Functionalized NGO-AgNPs-PEG nanocomposite was washed and purified by using ultrapure water. Finally, doxorubicin (DOX) was coupled to the nanosystem by adding the nanocomposite to an aqueous solution of the drug and keeping the final mixture in a shaking incubator at 28 °C for 24 h. Then, dialysis and lyophilization were carried out. DOX interacts with the nanosystem through a coordinated covalent bond to NGO-AgNPs-PEG. Its aromatic rings form π-π interactions, and the drug also establishes hydrogen bonds, with partially reduced graphene oxide. In addition, DOX, which has a positive zeta potential (+3.2 mV), appears to form electrostatic interactions with NGO-AgNPs-PEG, which have a negative zeta potential. Electrostatic interactions are formed between the DOX ionized amine group and the nanosystems carboxylates. The loading of DOX at the NGO-AgNPs-PEG increased the zeta potential from −11.7 mV (NGO-AgNPs-PEG) to −8.9 mV (NGO-AgNPs-PEG-DOX). This aspect is fundamental, considering that the negative zeta potential value maximizes the circulation time, circumvents fast RES clearance, provides higher physiological stability of nanocomposites and facilitates blood brain barrier crossing, contributing thus to an optimum enhanced permeability and retention (EPR) effect. Overall, the loaded anticancer drug has its pharmacological efficacy boosted, comparing to the free DOX. By keeping the concentration of DOX at 50 µg/mL, the loading capacities of NGO-AgNPs-PEG were evaluated. Increasing concentrations of NGO-AgNPs-PEG nanocomposites were assayed (1, 10, and 100 µg/mL), also including a test for 10 µg/mL of NGO. The obtained results were 194, 218, 73, and 198%, respectively. Thus, the authors opted to use throughout the work the concentration of 10 µg/mL of NGO-AgNPs-PEG to prepare NGO-AgNPs-PEG-DOX nanocomposite. The release of the drug by the nanosystem, at the two different pH 7.4 and 5.4, was studied. It was concluded that a higher rate of drug release at acidic pH while, at both pH, it was possible to observe a controlled release of doxorubicin up to 120 h. The authors suggested that acidic pH enhances the release of doxorubicin since it would weaken the hydrogen bonds between doxorubicin and GO and, also, reduce electrostatic interactions between the doxorubicin amino group and the NGO-AgNPs-PEG carboxylic acids. These observations reveal great interest, given that the tumor microenvironment also has an acidic pH, thus being able to trigger the release of the drug. This constitutes an advantage in directing the drug to the target. The cytotoxicity of the nanosystem was evaluated on HeLa cell line (cancer cell line) and HaCaT cell line (human keratinocyte cell line). Although the NGO-AgNPs-PEG-DOX had a less damaging effect on cancer cells of the HeLa cell line (only about 41.56% cell viability) compared to free doxorubicin (23.82%), it showed less cytotoxicity in the normal HaCaT keratinocyte cell line (cell viability of 79.17 and 46.76% for NGO-AgNPs-PEG-DOX and free doxorubicin, respectively). In conclusion, this nanosystem showed lesser harmful effect on normal cells compared to free doxorubicin. Since DOX can produce severe side effects during treatment because it cannot differentiate between normal cells and cancer cells, the AgNPs’ nanocarrier of DOX in the form of NGO-AgNPs-PEG-DOX nanoplatform proved to be advantageous, compared with the use of free DOX.

Zeng et al. (2018) [74] assembled AgNPs coated with graphene oxide (Ag-NGO) as doxorubicin nanocarriers. First, the NGO was synthesized using graphite powder added to sulfuric acid in an ice bath, and then by adding potassium permanganate slowly under mild stirring. The mixture was kept stirring at 50 °C for 3 h, and after some other simple procedures, when the solution became bright yellow, hydrogen peroxide was added. The mixture was washed with hydrochloric acid twice, suspended in nitric acid, and heated at 80 °C for 24 h. Finally, it was washed with deionized water until the pH of the mixture was neutral, sonicated for 2 h, and filtrated using 0.22-µm filter membrane to obtain an NGO solution. Then, to prepare the Ag-NGO nanosystem, a mixture of silver nitrate and NGO solution was stirred for 20 min under cooling temperatures. The reductant of silver ions was sodium borohydride, added drop by drop, until the mixture became yellow. The AgNPs produced in the NGO matrix were determined to be of about 40 nm in size, by DLS. After proper washing with deionized water, the nanocomposite Ag-NGO was mixed 10 min with a solution of the anticancer DOX, and centrifuged to remove the supernatant, obtaining finally the nanocarrier Ag-NGO-DOX. The drug loading amount was calculated as 8 wt%. The solubility and the bioavailability of the Ag-NGO-DOX nanosystems was assured by the graphene oxide present at the surface of AgNPs, which confers the surface with hydroxyl, carboxyl, and epoxide functional groups, improving those properties. Also, NGO enables functionalization with other molecules, such as anticancer drugs and targeting ligands, since it is negatively charged on the surface of AgNPs, giving them negative zeta potential. Doxorubicin interacts with graphene oxide through π-π interactions.

In addition to the authors studying the Ag-NGO nanosystem as a successful nanocarrier of DOX, they also assayed its potential for being theragnostic, as nanoprobes for general intracellular biosensing. The Ag-NGO nanosystem was assayed as a biosensing nanoprobe through surface-enhanced Raman scattering (SERS). SERS is a surface-sensitive phenomenon, in which Raman signals of the adsorbed molecules at the surface of SERS-active substrate can be highly intensified [83]. In the work of Zeng et al., the surface enhancement for biosensing was tested by incubating HepG-2 cancer cells with Ag-NGO for 24 h. After proper sample pre-treatment for SERS monitoring, by using an appropriate laser wavelength to originate molecular resonance (633 nm laser of 12.5 mW), the authors were able to detect biomolecules inside the cells. Yet, biosensing information was not specific, but general, since the Ag-NGO nanoprobe was not targeting for a specific analyte.

**Figure 6:**
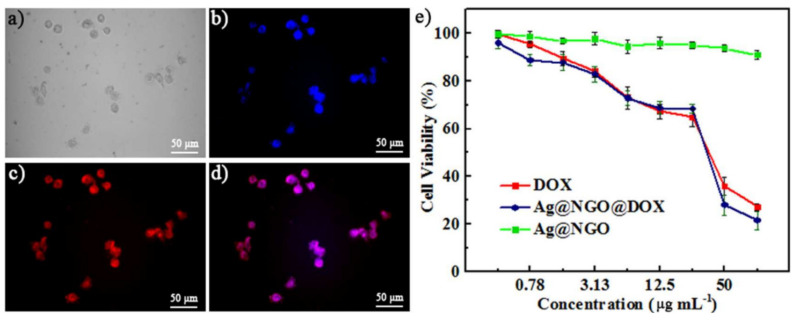
Fluorescence microscopy images of cancer cells HepG-2 after incubation with Ag-NGO-DOX (12.5 µg/mL): (**a**) bright field; (**b**) blue emission (λ_ex_ = 360 nm ± 20 nm) of the cell nucleus after staining with 4′,6-diamidino-2-phenylindole (DAPI); (**c**) red fluorescence emission of DOX inside the cells, by excitation at λ_ex_ = 490 ± 20 nm; (**d**) overlapping of (**b**,**c**), showing that DOX reached the cancer cells’ nucleus; (e) MTT assay viability results of HepG-2 liver cancer cells after incubation with different concentrations (0, 0.78, 1.56, 3.13, 6.25, 12.5, 25, 50, and 100 µg/mL) of free DOX, Ag-NGO, and Ag-NGO-DOX. Reproduced from [74], with permission from American Chemical Society, 2018.

Regarding the cytotoxic performance of the proposed Ag-NGO-DOX nano-assembly, it was dependent on the acidic intracellular cancer medium and the surrounding tumor microenvironment. As mentioned before, in previous discussed works, the interaction between doxorubicin and graphene oxide is sensitive to the pH of the medium, and the acidic conditions associated with the tumor microenvironment triggers the drug’s release from the nanocarrier (Figure 6). Figure 6d shows that the Ag-NGO-DOX nanocarrier was able to successfully deliver the loaded drug and released it at proper location, due to the intracellular acidic pH responsive release of DOX, making possible the drug to reach the cancer cells’ nucleus.

Following this, the authors conducted some cytotoxicity assays, by 3-(4,5-dimethylthiazol-2-yl)-2,5-diphenyltetrazolium bromide (MTT) assay, on the HepG2 cancer cell line and on a normal HEK293 cell line. In the normal cell line, only Ag-NGO nanoparticles were tested, showing low toxicity (about 75% cell viability at the maximum concentration tested, 100 µg/mL). On the HepG2 cell line, Ag-NGO nanoparticles, Ag-NGO-DOX nanosystem, and free DOX were tested. Regarding Ag-NGO, it was found that they were not toxic for this cell line (cell viability about 90% for the maximum concentration tested, 100 µg/mL, deducted by observation of Figure 6e). In the work, the precise values of the cell viabilities were not found. The authors mention that the good biocompatibility of Ag-NGO could be attributed to the NGO coating shell. In the case of Ag-NGO-DOX, with the same amount of DOX, cell viability was slightly lower compared to treatment with the free drug (around 20% for Ag-NGO-DOX and approximately 25% for free doxorubicin, deducted by observation of Figure 6e). This observation seems to show that the Ag-NGO nanosystem enhances the drug delivery, in addition to being biocompatible. Moreover, it has the advantage that it could also be used in SERS-based biosensing.

Leaves of *Butea monosperma*, rich in polyphenols and proteins, were used to produce AgNPs by biosynthesis for further use as carriers of doxorubicin. In the work developed by Patra et al. [41] (2015), the authors prepared an aqueous extract of fresh leaves of *Butea monosperma*, by initially washing with double distilled water followed by sterile Millipore water. The leaves were kept in ultrapure water and heated for 3 min under microwave irradiation. This mixture was kept overnight under stirring conditions at room temperature and then centrifuged at 7000 rpm at 10 °C for 30 min. Next, by using the prepared extract and silver nitrate, the AgNPs were synthesized. These had a zeta potential of −14.7 ±0.5 mV, and TEM images revealed large spherical nanoparticles (20–80 nm) with the presence of a few triangular b-AgNPs, most probably related with a slowly growth process of AgNPs promoted by weaker reducing agents in the *B. monosperma* leaf extracts. Finally, doxorubicin was added to the AgNPs solution and stirred vigorously for 60 min. The nanocomposites DOX-b-AgNPs were purified by ultracentrifugation at 14,000 rpm at 15 °C for 30 min. *Butea monosperma* leaf extract was the source for the chemical substances with reducing, stabilizing, and capping agent properties for the AgNPs synthesis. Specifically, molecules with hydroxyl groups (polyphenols) and proteins present in the extract were responsible for the reduction of silver ions. Additionally, the authors confirmed that the capping of the AgNPs with the proteins caused the disappearing of the typical SPR band. In the work, the authors pointed out that doxorubicin molecules appeared to interact with AgNPs through coordinated bonds with their hydroxyl groups and electrostatic interactions, since the drug had a positive zeta potential and the AgNPs had a negative zeta potential. The drug loading amount was estimated as 13%. The cytotoxicity of DOX-b-AgNPs was assessed by monitoring the inhibition of cancer cells proliferation by MTT assay, on the cancer cell lines MCF-7 and B16F10, after incubation with free doxorubicin and DOX-b-AgNPs (Table 1). It was found that the DOX-b-AgNPs nanosystem caused greater inhibition of the proliferation of cancer cells B16F10 of murine melanoma, and MCF-7, than free doxorubicin. The authors did not provide precise values for the measured % of cell viabilities. This nanosystem showed better therapeutic efficacy towards these cancer cell lines compared to free doxorubicin at the same concentration, probably due to a greater internalization of doxorubicin when coupled to AgNPs, as was verified through fluorescence microscopy in B16F10 cells. Nevertheless, it would be relevant to perform cytotoxicity tests on normal cells, to verify whether this nanoconjugate is less harmful to these cells.

### 4.3. Folic Acid Anticancer Drug

In 2012, folic acid was used as capping agent for the synthesis of AgNPs by Wang et al. [75], which synthetized these to be used as nanocarriers of doxorubicin (FA-AgNPs-DOX). First, folic acid (capping agent) and ascorbic acid (reducing agent) were added simultaneously to a pre-heated solution of silver nitrate, and the pH was tuned between 11−12 by using sodium hydroxide. The AgNPs (FA-AgNPs) were synthesized after 2 min of boiling. The as-synthesized AgNPs presented an average size of 23 nm and an UV-vis absorption maximum at 409 nm. Subsequently, doxorubicin was added to the FA-AgNPs and the mixture was stored at room temperature for 3 h, completing the formation of DOX-FA-AgNPs. Next, the DOX-FA-AgNPs were purified by centrifugation at 8000 rpm for 15 min. Folic acid (Figure 3H) interacts with the nanoparticles through an Ag-N bond. The DOX molecules were coupled to the coated nanoparticles through electrostatic interactions between the negative charges of the folic acid molecules, due to the carboxylic acids, and the positive charges of the drug molecules. As already stated before, the folic acid receptor is over-expressed in some cancers, while in normal tissues has a limited distribution. So, human carcinoma cells derived from the nasopharynx (KB cancer cells) were used in a viability assay, expecting good results since they have a high expression of folate receptors. Simultaneously, the SK-BR3 breast cancer cell line was used as control since it has a low expression of folic acid receptors. Yet, the authors affirm that free doxorubicin caused equal cytotoxic effect on cells lines KB and SK-BR3, and they observed higher cytotoxicity in the KB cell line than with the DOX-FA-AgNPs nanocarrier. However, the major advantage of the described nanosystem DOX-FA-AgNPs was revealed when assaying both KB cells and SK-BR3 cells incubated with DOX-FA-AgNPs, in which the nanosystem originated a higher cytotoxic effect in KB cells compared to SK-BR3 cells. Hence, it was demonstrated the higher cytotoxic effect of the nanoconjugates more oriented towards cells that overexpress the folate receptor than the free drug. This strategy is a good example of active targeting of NPs, which shows benefits in reducing systemic toxicity, as previously discussed. In this sense, it would be interesting to have tested the cytotoxicity of FA-AgNPs-DOX in normal cells, to assess their greater selectivity for cancer cells.

### 4.4. Alendronate Anticancer Drug

**Figure 7:**
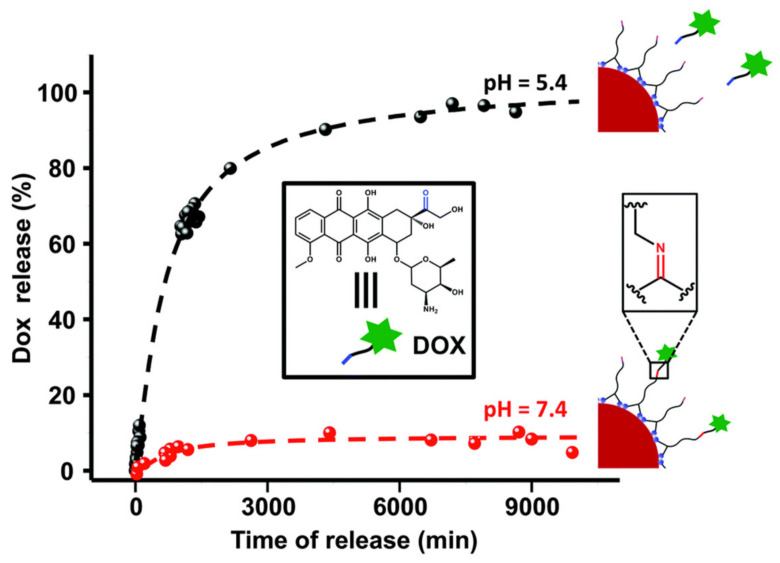
Influence of pH (37 °C, pH 7.4 (red curve) and pH 5.4 (black curve)) on DOX release profiles from DOX-Ald@AgNPs. Reproduced from Ref. [76] with permission from The Royal Society of Chemistry, 2021.

As mentioned above, doxorubicin can cause severe adverse effects, such as cardiomyopathy. Two strategies can be used to reduce the dose and therefore its toxicity: improving its cellular uptake or administering other drugs simultaneously, thus exploiting a synergistic effect [76]. It was observed by Benyettou et al. [76] that doxorubicin is more effective against breast cancer when it is given in combination with alendronate (Ald), a bisphosphonate. In 2015, Benyettou et al. [76] resorted to chemical synthesis to produce AgNPs coupled with alendronate (Figure 3C), which in turn was linked to doxorubicin. The synthesis of the dual-nanocarrier was initiated with the addition of an aqueous solution of silver nitrate to an aqueous solution of alendronate. This mixture was next heated in the microwave, at 70 °C and pH 4, for 15 min, with stirring. The resulting suspension was dialyzed for two days. The as-synthesized AgNPs had 11 nm of diameter and presented an UV-vis absorption maximum at 412 nm. Subsequently, the alendronate-conjugated AgNPs were added to an aqueous mixture containing doxorubicin, EDC, and NHS (N-hydroxysuccinimide). The mixture was stirred for 24 h at room temperature and then dialyzed for 48 h. The DOX-Ald@AgNPs were separated by filtration. The zeta potential of DOX-Ald@AgNPs was −37 mV at pH = 7.4, due to the negative charges conferred by alendronate molecules. According to the authors, alendronate interacts with AgNPs through bisphosphonate groups, negatively charged, by chelation-based mechanisms. In fact, Ald does not act as reducing agent in the AgNPs synthesis, but rather a nucleation site that promotes the development of small nanoclusters of silver to AgNPs. The authors pointed out that the microwave radiation was responsible for the formation of solvated electrons from water molecules, which next reduce Ag+ to Ag^0^. Then, the bisphosphonates groups of Ald form complexes with silver (I) and act as nucleation sites, controlling the growth and size of the AgNPs, while at the same time providing stability by chelation. As regards the reaction between Ald and DOX, carbonyl groups of DOX bind to the free amine of alendronate, forming imine. The mass of Dox attached at the DOX-Ald@AgNPs was estimated by thermogravimetric analysis (TGA), revealing an average of 682 Dox molecules per each particle. Considering that DOX conjugation depends on Ald conjugation on the AgNPs, and that an average of 4115 Ald molecules per particle was found, the conjugation efficiency of Dox was estimated at ~17%. The release of the drug DOX was dependent on the surrounding pH, that is, it was promoted by acidic pH, normally verified in the tumor microenvironment. The acidic pH promoted the hydrolysis of the imine bond between doxorubicin and alendronate. To support this fact, two different conditions were tested, an acidic aqueous sample (pH 5.4) and a physiological aqueous sample (pH 7.4) of the DOX-Ald@AgNPs. Over the same period, only ~9% of doxorubicin was released at pH 7.4, while with pH 5.4, 95% of this drug was released (Figure 7).

The cytotoxic effect of this nanoconjugate and free doxorubicin was evaluated on HeLa cancer cells. The nanocarrier DOX-Ald@AgNPs originated an IC_50_ = 0.1 µM, lower than that of doxorubicin alone (3 µM, Table 1), and therefore the nanosystem composed by AgNPs coupled to alendronate and doxorubicin revealed a major synergistic effect against the HeLa cell line, in in vitro assays. In this work, it would have been interesting to conduct studies on a normal cell line to see if there was less toxicity in healthy cells, to assess the selectivity of DOX-Ald@AgNPs nanocarrier.

### 4.5. Epirubicin Anticancer Drug

The drug epirubicin (Figure 3D) belongs to the anthracycline family of anticancer agents used to treat breast, lung, and liver cancer. It has several hydroxyl groups, presenting thus useful reducing properties [71]. This drug was another example found in the literature, which was assessed for its anticancer effect when in conjugation with AgNPs acting as nanocarriers. In the work of Ding et al. [71] (2019), it was chemically synthesized AgNPs coupled with epirubicin (EPI), by adding the anticancer drug to a boiling solution of silver nitrate, maintained at 120 °C for 1 h. The purification of the nanoconjugate was accomplished by dialysis in ultra-pure water for 48 h (to remove unbound EPI), followed by centrifugation at 10,000× *g* for 30 min. The as-synthesized AgNPs showed an average diameter of 36 nm. The drug itself was used as a reducing agent. According to this work, considering that epirubicin was subjected to 120 °C during 1 h, apparently the chemical synthesis of the EPI-AgNPs did not cause any chemical modification to the drug that would impair its pharmacological activity. The authors did not mention in the work what type of interaction was established between the drug and the nanoparticles. However, given the similarity between epirubicin and doxorubicin, it can be assumed that drug molecules established coordinated covalent bonds with AgNPs, possibly through their hydroxyls groups that were not oxidized when the reduction of AgNPs occurred. To assess the anticancer activity of EPI-AgNPs, the HepG2 cell line was chosen, and cytotoxicity effects obtained by the MTT assays revealed that the IC_50_ of the nanoparticles coated with epirubicin was in fact higher than the IC_50_ of the free drug (1.92 µg/mL and 0.11 µg/mL, respectively). However, in theory, this nanocarrier could reduce the incidence of adverse effects, since it would be retained in locations close to the tumor due to the EPR effect, thus reducing its exposure to normal tissues, as explained in Section 3.4. Indeed, the authors claimed that the nanosystem provided major advantages considering the severe side effects and toxicity observed with the large doses of the drug epirubicin required by the treatments. Due to the EPR effect of the AgNPs, only the tumor tissues would be most targeted by the nanosystem, and hence, the toxicity of the treatment on healthy cells would diminish. However, this should be confirmed using tests in normal cells and, later, in vivo.

### 4.6. Paclitaxel Anticancer Drug

Paclitaxel (Figure 3E) is an anticancer drug used to treat various solid tumors, namely lung, breast, and ovarian cancers, though it has a high incidence of adverse effects (fever, anemia, bleeding, hair loss, skin rash, among others) and low solubility in aqueous media [77,84].

**Figure 8:**
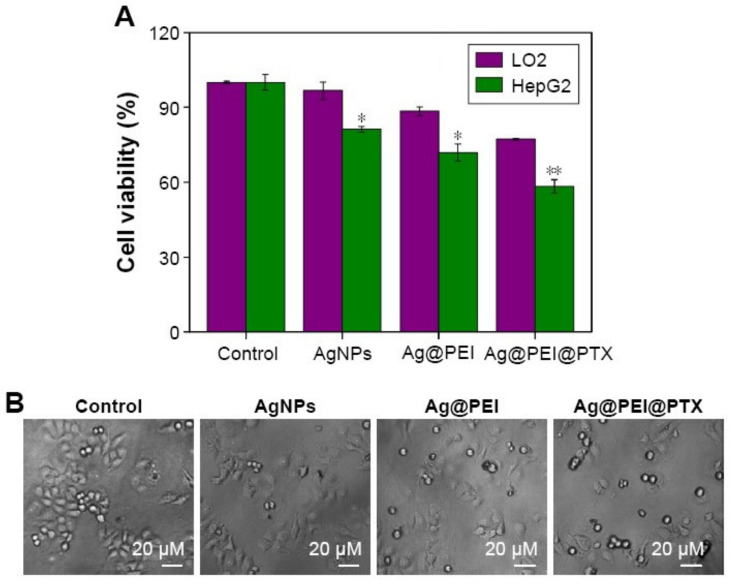
Influence of AgNPs, Ag@PEI, and Ag@PEI@PTX on the growth of HepG2 cells: (**A**) at concentrations of 2.5 μg/mL, on the cell viabilities of hepatocarcinoma HepG2 and normal human liver LO2 cell lines, for 24 h by MTT assay; and (**B**) respective morphological changes. Statistical significance analyzed at different levels: * *p* < 0.05, ** *p* < 0.01. Reproduced from Ref. [77], originally from Dove Medical Press Ltd.

Polyethyleneimine (PEI)-modified AgNPs functionalized with paclitaxel (PTX) (Ag@PEI@PTX), were assembled in 2016, by Li et al. [77], through chemical synthesis. For the AgNPs synthesis, ascorbic acid solution was used as reducing agent, added dropwise, under continuous magnetic stirring, to a silver nitrate solution, for 2 h at room temperature. Later, an amount of PEI and PTX was added to the as-prepared AgNPs with ultrapure water, leading to the formation of the Ag@PEI@PTX complex. The purification of the nanocarrier was made overnight by dialysis, after that the Ag@PEI@PTX suspension was sonicated, and then filtered (0.2 µm). The as-prepared Ag@PEI@PTX showed a minimum diameter of 2 nm. The authors did not perform studies to determine what type of interaction was established between the drug and the nanoparticles. The zeta potential value of Ag@PEI@PTX was 23 mV, and so, being positive it promoted easier passage through the cells’ membranes. The polyethyleneimine (Figure 3I) used to functionalize the nanoparticles is a polycationic polymer and, consequently, conferred a positive charge to the nanocarrier. Cell viability assays were performed on hepatocarcinoma HepG2 and normal human liver LO2 cell lines (Figure 8). The Ag@PEI@PTX nanosystem inhibited the proliferation of HepG2 cells and showed low cytotoxicity in LO2 cells. In fact, in Table 1, it can be verified that the cell viability of Ag@PEI@PTX towards HepG2 cells and LO2 cells was about 58.32 and 77.21%, respectively. In this work, the authors did not carry out cell viability tests with free paclitaxel, so it was not possible to draw conclusions about whether this nanosystem is more advantageous or not than the free drug.

### 4.7. Imatinib Anticancer Drug

**Figure 9:**
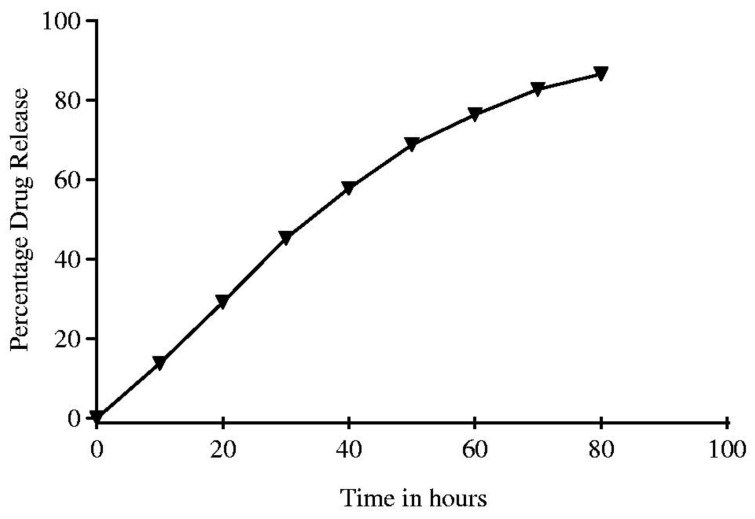
Profile of in vitro release of IMAB from IMAB-AgNPs, at 80 h of contact time in phosphate buffer (pH = 7.4), at 37 °C. Reprinted from [40], with permission of the publisher Taylor & Francis Ltd., 2021.

Imatinib (Figure 3F) is an anticancer drug also used to treat various solid tumors, such as skin and stomach cancers, as well as certain types of leukemia [85]. However, it has a high risk of adverse effects, which include bleeding, coughing, difficulty breathing, muscle pain, fatigue, among others [40,85]. AgNPs coupled with imatinib (IMAB), through biosynthesis, were synthesized in 2017 by Shandiz et al. [40]. Briefly, *Eucalyptus procera* extract was prepared and added to an aqueous solution of silver nitrate, under continuous stirring, till an intense brown color appeared, showing that it was indicative of the formation of AgNPs. The leaves’ extract of *E. procera* behaved as a reducing and capping agent, probably due to the high content in alcohols and phenols. The purification of the as-synthesized AgNPs was carried out by centrifugation at 13,000× rpm for 20 min. The obtained AgNPs presented an average size of 20 nm and a SPR band with maximum absorption at 438 nm. As a last step, imatinib was added to AgNPs, under stirring at room temperature for 72 h, resulting in imatinib-loaded AgNPs (IMAB-AgNPs). The IMAB-AgNPs were separated by centrifugation at 10,000× rpm for 60 min at 25 °C and washed twice with double distilled water. The authors executed some studies to test the influence of the amount of the drug, used in the synthesis, in the loading capacity of the nanocarrier, and found out that for 10 mg and 20 mg of IMAB, the percentages of drug loading were 83.45 and 95.30%, respectively. It was not mentioned in the work what type of interaction was established between the drug and the nanoparticles. One could hypothesize that imatinib interacts with the capping molecules originated from the complex natural product extract. However, this hypothesis was not confirmed and should be verified. Some drug release assays were also performed (Figure 9), and the authors concluded that the IMAB release profile could be divided into two stages. In the first stage, till ~40 h, there was a burst release of the drug (29.22 ± 3.01% in the first 20 h and ~57.89 ± 1.48% in the initial 40 h), and in the second stage, the drug was released gradually till a release of 86.56 ± 2.04% at 80 h of contact time in a releasing simulating medium of phosphate buffer, pH 7.4, 37 °C temperature.

**Figure 10:**
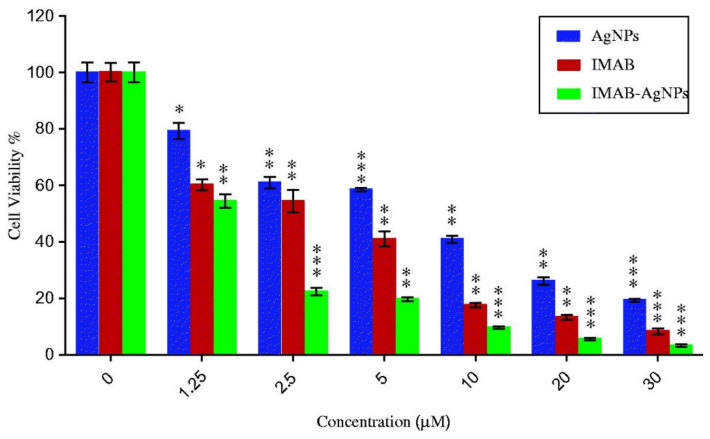
Influence of AgNPs, free IMAB, and IMAB-AgNPs on MCF-7 cancer cells, at different concentrations (0, 1.25, 2.5, 5, 10, 20, and 30 µM) and after incubation of 24 h. Statistical significance analyzed with Student’s t-test at different levels: * *p* < 0.05, ** *p* < 0.01 and *** *p* < 0.001. Reprinted from [40], with permission of the publisher Taylor & Francis Ltd., 2021.

Cytotoxicity MTT-based assays of AgNPs, free drug, and IMAB-AgNPs were performed against the MCF-7 breast cancer cell line (Figure 10). In Figure 10, one can observe that with increasing concentrations of IMAB-AgNPs, the cytotoxicity was also increasing, being more pronounced for higher values of concentrations. The IMAB-AgNPs showed greater cytotoxicity in MCF-7 cells than the free drug (the IC_50_ determined was about 1.69 µM and 3.02 µM, respectively). As additional information, the calculated IC50 of AgNPs was 9.63 µM. In this work, it would be important to include studies with normal cells to fully understand the impact of IMAB-AgNPs on cancer and healthy cells.

### 4.8. Gemcitabine Anticancer Drug

**Figure 11:**
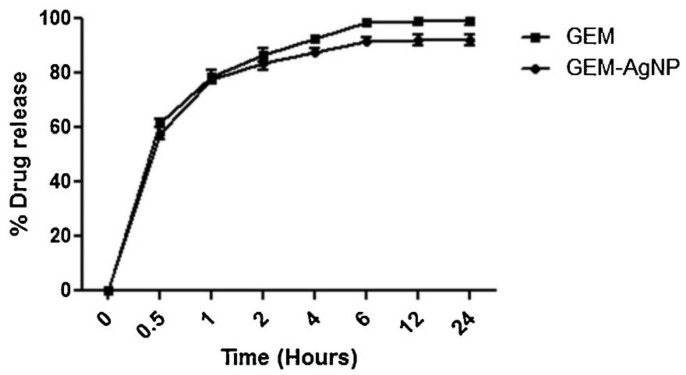
Profiles of GEM release, free in solution and from GEM-AgNPs, in a phosphate buffer solution (pH = 7.4), for 24 h. Reprinted from [59], with permission from Elsevier, 2021.

AgNPs were also exploited as nanocarriers of the anticancer drug gemcitabine in 2020 by Karuppaiah et al. [59]. Gemcitabine (Figure 3G) is an anticancer drug used in the treatment of various cancers, namely pancreas, lung, and breast cancers. Gemcitabine can cause side effects like low blood cell counts, fever, liver problems, and fluid build-up in or around the lungs [86]. To synthesize the AgNPs, the authors opted for a procedure involving a silver nitrate solution being added dropwise to an ice-cold sodium borohydride solution, which is a very strong reducing agent, followed by magnetic stirring until a bright yellow color was obtained. To prevent the AgNPs’ aggregation and enhance their stability, PVP was used as a capping agent. After purification by centrifugation at 13,000× rpm for 45 min, the as-prepared AgNPs presented an average particle size of 9.16 nm, and an UV-vis absorption maximum at 395 nm, which was in close agreement with the small size measure by TEM. Finally, gemcitabine (GEM) was added to the AgNPs suspension and stirred at room temperature for 24 h, forming GEM-AgNPs. Again, centrifuging at 10,000× rpm for 15 min allowed to separate GEM-AgNPs. GEM was coupled to the surface of AgNPs through electrostatic interactions since the drug is positively charged and the nanoparticles were negatively charged (zeta potential = −46.7 ± 0.25 mV), due to the PVP capping. The adsorption efficiency was calculated as 75.6%. Some drug release assays of GEM-AgNPs were also performed, including a comparison with the profile obtained for a solution of free GEM. Both solutions presented a similar release profile (Figure 11), but the authors stated that a burst release in a 4-h period was observed reaching 92.5 ± 1.75% for GEM solution and 87 ± 1.52% for GEM-AgNPs, and that additionally, the maximum drug release was achieved at the end of the 24 h, for both free GEM and GEM-AgNPs (fixed at 98.9 ± 1.53%, and 91.5 ± 1.45%, respectively).

**Figure 12:**
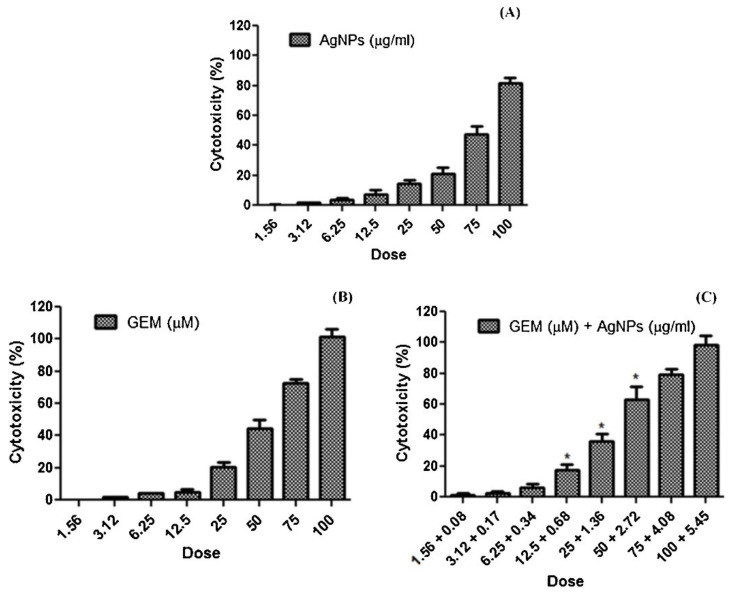
Cytotoxicity studies with AgNPs (**A**), free GEM (**B**), and GEM-AgNPs (**C**) against the breast cancer cell line MDA-MB-453, at concentrations of 1.56, 3.12, 6.25, 12.5, 25, 50, 75, and 100 µM. (*)—Statistical significance analyzed at the level *p* < 0.05. Reprinted from [59], with permission from Elsevier, 2021.

The authors also conducted cytotoxicity studies with AgNPs and GEM-AgNPs on the breast cancer cell line MDA-MB-453. They found that up to a concentration of 6.25 µg/mL (Figure 12A), AgNPs did not produce significant cytotoxicity to the inhibition of the breast cancer cell line MDA-MB-453. Aiming at testing the possible synergistic effect between AgNPs and Gem in the form of GEM-AgNPs, different concentrations of gemcitabine conjugated to AgNPs were prepared, whose maximum concentration was fixed at 5.45 µg/mL, representing this as a AgNPs concentration with proved non-cytotoxic effects (Figure 12C). When the authors compared the results obtained with GEM-AgNPs, with the results from similar doses of free GEM (Figure 12B), they noticed a significant improvement in cytotoxicity (%) for the concentrations of 12.5, 25.0, and 50.0 μM. In fact, the obtained IC_50_ of the GEM-AgNPs was lower (37.64 µM, Table 1) than that of the free drug (56.54 μM).

Hence, the conjugation of GEM with non-cytotoxic concentrations of AgNPs showed a greater cytotoxic effect on breast cancer cells MDA-MB-453 compared to the free drug, demonstrating the synergism between gemcitabine and AgNPs. This work proved that the therapeutic combination can be advantageous in that it allows the use of lower doses of chemotherapeutic agents, which results in the reduction of the incidence of adverse effects and improved efficiency. Also, the use of non-cytotoxic concentrations of AgNPs might reduce damage to normal cells, but it would be interesting to have performed cytotoxicity assays of GEM-AgNPs on normal cell lines and, later, in vivo.

## 5. Conclusions

Silver nanoparticles can be very useful in the transport of anticancer drugs, overcoming some of the difficulties of conventional therapies. The strategy of coupling anticancer agents to silver nanoparticles seems to have emerged in the last decade, since the oldest paper that was found in the databases dates to 2012. In fact, as it was possible to confirm, the selected papers are quite recent (between 2012 and 2020), thus showing that nanotechnology has made many advances in recent years. Five of the eleven studies are based on the use of the anticancer drug doxorubicin, possibly because it is used to treat many cancers (breast, bladder, ovaries, thyroid, lungs, etc.) and because it has serious adverse effects, which could be reduced using nanocarriers.

In addition, there was a relatively small number of studies focusing on the synthesis of this type of nanosystem. The biosynthesis of AgNPs has been gaining relevance as it is a strategy that does not use chemical reagents and is simple and environmentally friendly. Of the total number of papers presented, three used this method.

The analyzed articles showed that silver nanoparticles can have a synergistic effect with anticancer drugs, allowing the use of lower doses. Therefore, these nanocarriers also provide less toxicity in healthy cells, potentially reducing the side effects caused by anticancer agents. Moreover, the EPR effect and active targeting can also contribute to greater selectivity for tumors. However, silver is not extensively used in drug delivery nanosystems since there are some concerns regarding the toxicity and stability. Instead, it is being replaced with gold or other nanomaterials, such as liposomes.

It is expected, in the coming years, that this research area will be expanded, with a greater diversity of anticancer drugs to be used and greater knowledge of the in vivo behavior of the nanosystems, in terms of absorption, distribution, metabolism, excretion, and toxicity.

## Figures and Tables

**Table 1:** Anticancer drugs coupled silver nanoparticles main physicochemical characteristics and their cytotoxic effects in in vitro cancer models.

Anti-Cancer Drug Capped AgNPs							In Vitro Assays		
Anticancer Drug	Method of Synthesis	Functionalization Ligand	Synthesis Step	Reagents	SPR Band (nm)	Size (nm)	Viability Test	Cancer Cell Line	Cell Viability (%)/IC_50_ (µg/mL)
Methotrexate [73]	Conventional heating	MTX	(i) AgNPs	NaBH_4_	394	DLS: 14.7 ± 2.7 HRTEM: 11.13 ± 2.27	FDA hydrolysis	HTC-116 colorectal cancer	
				Trisodium citrate dehydrate					88 µg/mL (12 h)
									38 µg/mL (24 h)
				AgNO_3_					23 µg/mL (48 h)
				NaOH					
			(ii) MTX solution	MTX	-			A-549 human lung carcinoma	≈86% (12 h)
				K_2_CO_3_					≈79% (24 h)
			(iii) AgNPs-MTX	MTX	405	DLS: 21.9 ± 8.7 HRTEM: 15.2 ± 3.9			≈32% (48 h)
				AgNPs					
Methotrexate [30]	Conventional heating	PVPK30	GO dispersion	Graphite	-	TEM: 22.3 ± 2.7	MTS	MCF-7 breast cancer	n/a
				H_2_SO_4_					
				KMnO_4_					
				H_2_O:H_2_O_2_ (3:1)					
			GO/AgNPs	GO dispersion	400			HepG2 liver cancer	n/a
				AgNO_3_					
				Glucose					
				Starch					
			MTX-GO/AgNPs	GO/AgNPs	-				
				EDC					
				MTX					
				PVPK30					
Doxorubicin [70]	Biosynthesis		GO suspension	KMnO_4_	-	HRTEM: 25	MTT	HeLa	41.56%
				H_2_SO_4_					
				H_2_O_2_					
			Aqueous extract	*Azadirachta indica*	-				
			NGO-AgNPs	Silver nitrate	436				
		mPEG-NH_2_		GO dispersion					
		DOX		Aqueous extract					
			NGO-AgNPs-PEG	mPEG-NH_2_	-				
				NGO-AgNPs					
				EDC					
				NHS					
			NGO-AgNPs-PEG-DOX	DOX	-				
				NGO-AgNPs-PEG					
Doxorubicin [74]	Conventional heating		NGO	Graphite powder	-	-	MTT	HepG2 liver cancer	n/a
				H_2_SO_4_					
				KMnO_4_					
				H_2_O_2_					
		GO		HNO_3_					
		DOX	AgNPs-NGO	AgNO_3_	400				
				NGO		TEM: 20			
				NaBH_4_		DLS: 40			
			AgNPs-NGO-DOX	AgNPs-GO	-	-			
				DOX					
Doxorubicin [41]	Biosynthesis	*Butea monosperma* leaf extract	Aqueous extract	*Butea monosperma*	-	-	MTT	B16F10 murine melanoma	n/a
			AgNPs	AgNO_3_	440–475	TEM: 50			
				Aqueous extract		DLS: 82.17 ± 1.8		MCF-7 breast cancer	n/a
			AgNPs-DOX	DOX	-	DLS: 107.32 ± 2.1			
				AgNPs					
Doxorubicin [75]	Conventional heating		FA-AgNPs	AgNO_3_	409	TEM: 23 ± 2	MTT	KB human carcinoma	n/a
				FA					
		Folic acid		Ascorbic acid					
		DOX		NaOH					
			FA-AgNPs-DOX	FA-AgNPs	-	-			
				DOX					
Doxorubicin Alendronate [76]	Microwave		ALD-AgNPs	AgNO_3_	412	SEM: 11	Resazurin	HeLa	0.1 µM
				ALD		TEM: 11			
		ALD				DLS: 20			
		DOX	DOX-ALD-AgNPs	ALD-AgNPs	-	-			
				DOX, EDC and NHS					

Epirubicin [71]	One-pot synthesis	Epirubicin	Epirubicin-AgNPs	AgNO_3_	-	TEM: 36	MTT	HepG2 liver cancer	1.92 µg/mL
				Epirubicin					
Paclitaxel [77]	RT		AgNPs	Vitamin C	-	-	MTT	HepG2 liver cancer	58.32%
		PEI		AgNO_3_					
		PTX	AgNPs-PEI-PTX	PEI	-	TEM: >2			
				PTX					
Imatinib [40]	Biosynthesis	*Eucalyptus procera* leaves extract	Aqueous extract	*Eucalyptus procera*	-	-	MTT	MCF-7 breast cancer	1.69 µM
			AgNPs	Aqueous extract	438	SEM: 60			
				AgNO_3_		TEM: 20			
						DLS: 63			
			IMAB-AgNPs	IMAB	-	SEM: 105–210			
				AgNPs		DLS: 148			
Gemcitabine [59]	RT		AgNPs	AgNO_3_	395 ± 0.28	DLS: 9.16 ± 0.28	MTT	MDA-MB-453 breast cancer	37.64 µM
				NaBH_4_					
		GEM	AgNPs stabilization	PVP	-	-			
		PVP		AgNPs					
			GEM-AgNPs	GEM	411 ± 0.57	DLS: 19.06 ± 0.50			
				AgNPs					

MTX: methotrexate; PVPK30: polyvinylpyrrolidone; GO: graphene oxide; EDC: N-(3dimethylaminopropyl-N-ethylcarbodiimide); mPEG-NH_2_: amine functionalized methoxypolyethylene glycol; DOX: doxorubicin; NGO-AgNPs-PEG: PEGylated silver decorated graphene nanocomposites; NHS: N-hydroxy succinimide; FA: folic acid; ALD: alendronate; PEI: polyethylenimine; PTX: paclitaxel; IMAB: imatinib; GEM: gemcitabine; PVP: polyvinyl pyrrolidone; DLS: Dynamic Light Scattering; SEM: Scanning Electron Microscopy; TEM: Transmission Electron Microscopy; HRTEM: High Resolution Transmission Electron Microscopy; FDA: Fluorescein diacetate; MTS: (3-(4,5-dimethylthiazol-2-yl)-5-(3-carboxymethoxyphenyl)-2-(4-sulfophenyl)-2H-tetrazolium); MTT: 3-(4,5-dimethylthiazol-2-yl)-2,5-diphenyltetrazolium bromide; n/a: not available.
